# Cefepime-Induced Delirium in a Patient With Chronic Kidney Disease: A Case Report

**DOI:** 10.7759/cureus.111418

**Published:** 2026-06-24

**Authors:** Helena Gomes, Miguel Pires, Catarina Freitas, Rodrigo M Freitas

**Affiliations:** 1 Psychiatry, Unidade Local de Saúde do Nordeste, Bragança, PRT; 2 Psychiatry and Mental Health, Unidade Local de Saúde da Guarda, Guarda, PRT; 3 Psychiatry, Unidade Local de Saúde de Barcelos/Esposende, Barcelos, PRT; 4 Psychiatry, Unidade Local de Saúde do Alto Minho, Viana do Castelo, PRT

**Keywords:** adverse drug reaction, cefepime-induced neurotoxicity, chronic kidney disease, delirium, renal impairment

## Abstract

Delirium is a frequent yet often underrecognised complication in hospitalised patients, particularly in those with renal impairment. Cefepime, a broad-spectrum cephalosporin, has been increasingly associated with neurotoxicity, manifesting as confusion, hallucinations, and agitation. This case report describes a 63-year-old woman with chronic kidney disease secondary to autosomal dominant polycystic kidney disease who developed acute confusional symptoms after initiating cefepime during treatment of a urinary tract infection associated with infected renal cysts. Following clinical improvement of the underlying infection, she developed temporal disorientation and visual hallucinations within 24-48 hours of cefepime initiation, which resolved within 24 hours of drug discontinuation. The Naranjo Adverse Drug Reaction Probability Scale classified the association as "probable" (score: 6). Although alternative contributors to delirium were present, including acute infection and renal dysfunction, the temporal relationship between cefepime exposure and symptom onset, together with rapid symptom resolution after withdrawal, supported cefepime-induced neurotoxicity as the most likely diagnosis. This case reinforces the importance of considering cefepime-induced neurotoxicity in the differential diagnosis of delirium, particularly in patients with renal dysfunction. Close clinical monitoring and early recognition are essential to prevent misdiagnosis and unnecessary interventions. Further studies are needed to refine risk assessment and explore safer therapeutic alternatives.

## Introduction

Delirium, or acute confusional state, is a neurocognitive disorder characterised by a disturbance in attention and awareness, with a rapid onset and a fluctuating course. It may include disorientation, language and memory impairments, altered visuospatial abilities, and perceptual changes. Diagnosis requires excluding other neurocognitive or psychiatric disorders that could better explain the symptoms, alongside evidence from history, examination, or tests indicating the disturbance is caused by a medical condition, substance use, or medication effects [1].

Delirium can result from multiple factors, including infections, metabolic issues, organ failure, and drugs - especially beta-lactam antibiotics, which are increasingly linked to its onset [2,3].

Cefepime, a broad-spectrum, fourth-generation cephalosporin, treats bacterial infections by inhibiting cell wall synthesis and is effective against both Gram-positive and Gram-negative bacteria, including some resistant strains [4]. Cefepime-induced neurotoxicity (CIN) is a well-documented adverse event, first reported by Wong et al. (1999) in haemodialysis patients [5]. Since 85% of cefepime is excreted renally, impaired kidney function - particularly reduced glomerular filtration - is the main CIN risk factor [6,7]. However, CIN has also occurred in patients with properly adjusted doses [8].

The pathophysiology of CIN is believed to involve concentration-dependent competitive inhibition of γ-aminobutyric acid (GABA) A receptors, as demonstrated in animal studies [6]. Cefepime crosses the blood-brain barrier and inhibits GABA-A receptors in the central nervous system, reducing inhibitory neurotransmission. Blocking GABA, the brain’s main inhibitory neurotransmitter, increases neuronal excitability, causing seizures, myoclonus, encephalopathy, and delirium [6,9]. Symptoms typically emerge within 1 to 10 days after drug initiation and resolve within 2 to 7 days of discontinuation [4].

Payne et al. (2017) reported cefepime neurotoxicity across varying renal functions, stressing careful monitoring despite dose adjustments [6]. Appa et al. (2017) emphasised frequent misdiagnosis as psychiatric or neurological disorders [10]. Therefore, CIN should be considered in the differential diagnosis of delirium, particularly in patients with renal impairment.

This case reviews the association between cefepime and delirium, underscoring the importance of clinical vigilance and careful monitoring.

## Case presentation

A 63-year-old female, married, with two children, working as a house cleaner, presented to the emergency department with dysuria, radiating pain from the right lumbar region to the right flank, macroscopic haematuria, and vomiting that had started earlier that day. Her history included autosomal dominant polycystic kidney disease (ADPKD) with chronic kidney disease (baseline serum creatinine of 3.2 mg/dL), arterial hypertension, migraines, renal lithiasis, and persistent depressive disorder. There was no relevant psychiatric family history.

The patient was on once-daily treatment with sertraline 50 mg, amitriptyline 10 mg, esomeprazole 40 mg, allopurinol 100 mg, ezetimibe/simvastatin 20/10 mg, erythropoietin 3000 IU, ferrous sulfate 329.7 mg, amlodipine 10 mg, and carvedilol 25 mg.

Initial laboratory tests revealed elevated creatinine (7.2 mg/dL), urea (140 mg/dL), and C-reactive protein (7.6 mg/dL) (Table 1). A computed tomography (CT) scan showed multiple spontaneously hyperdense cysts, suggesting haemorrhagic/protein content (Figure 1). The patient was admitted to the Internal Medicine Unit with a diagnosis of infected renal cysts and acute-on-chronic kidney dysfunction. Empirical treatment with intravenous ertapenem was initiated. On the sixth day of hospitalisation, urine culture identified *Enterobacter cloacae* complex susceptible to both ertapenem and cefepime. As the patient had shown clinical improvement, with resolution of fever and urinary symptoms, antimicrobial therapy was de-escalated from ertapenem to intravenous cefepime according to susceptibility testing results.

**Table 1:** Relevant laboratory findings during the diagnostic evaluation.

Parameters	Patient Values	Reference range
Serum creatinine	7.2 mg/dL	0.5-1.1 mg/dL
Baseline serum creatinine	3.2 mg/dL	0.5-1.1 mg/dL
Blood urea nitrogen	140 mg/dL	10-50 mg/dL
C-reactive protein	7.6 mg/dL	<0.5 mg/dL
Haemoglobin	8.2 g/dL	12.0-16.0 g/dL
Platelets	297 ×10⁹/L	150-450 ×10⁹/L
White blood cells	13.7 ×10⁹/L	4.0-11.0 ×10⁹/L
Neutrophils	91.6%	40-75%

**Figure 1:**
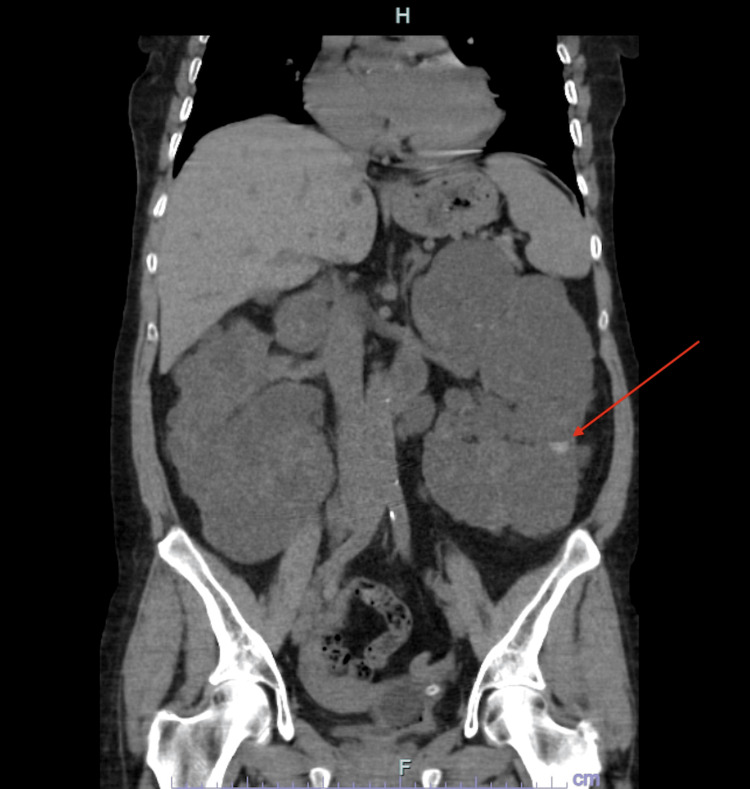
Coronal reformatted non-contrast abdominal CT. Coronal reformatted non-contrast abdominal CT demonstrating markedly enlarged bilateral kidneys with diffuse replacement of the renal parenchyma by innumerable cysts, consistent with autosomal dominant polycystic kidney disease. A spontaneously hyperdense cyst is present in the left kidney (red arrow), suggestive of proteinaceous content.

On the seventh day of hospitalisation, approximately 24 hours after cefepime initiation, the patient developed episodes of temporal disorientation. Head CT was unremarkable (Figure 2). On the eighth day, she exhibited visual hallucinations of insects, dust, hair in her coffee, and water on the floor, unperceived by staff. Soliloquies were also noted. Due to agitation, haloperidol 5 mg was administered intramuscularly. 

**Figure 2:**
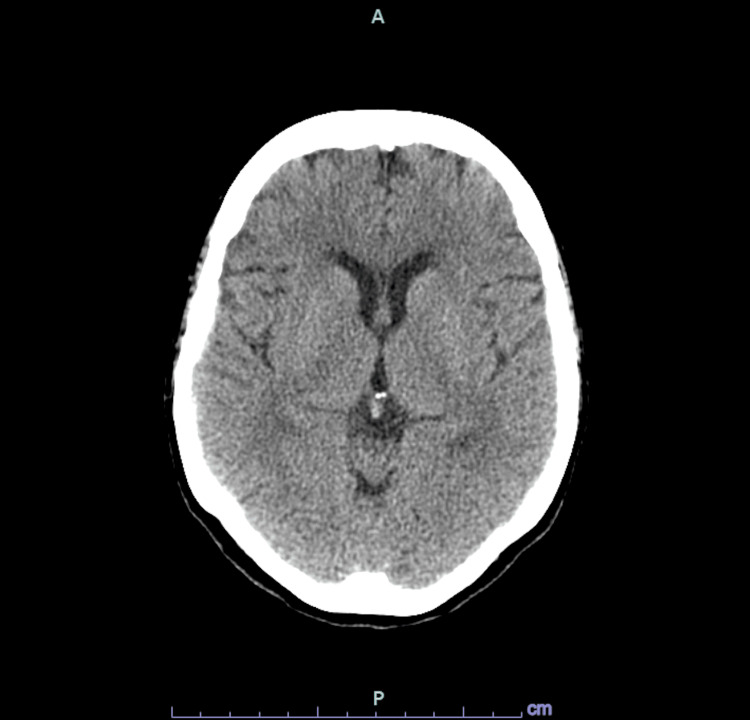
Non-contrast brain computed tomography. Non-contrast brain computed tomography demonstrating no acute intracranial abnormalities, with no evidence of haemorrhage, mass effect, or other structural lesions.

Although several factors potentially associated with delirium were present, including acute infection, anaemia, acute-on-chronic kidney dysfunction, polypharmacy, and recent exposure to ertapenem, cefepime-induced neurotoxicity was suspected because neuropsychiatric symptoms emerged only after cefepime initiation, following six days of ertapenem therapy without neurological manifestations. Cefepime was discontinued, and intravenous ertapenem was reintroduced.

On the following day, within 24 hours of cefepime withdrawal, psychiatric evaluation revealed no ongoing psychopathological alterations. The patient stated, “*I was confused, I did not know what I was doing.*” She was discharged on day 15 without the need for further psychiatric follow-up or medication adjustments.

## Discussion

This case highlights the importance of recognising CIN as a potential cause of delirium, particularly in patients with renal impairment. The patient developed acute confusional symptoms shortly after starting cefepime, which resolved rapidly after discontinuation, supporting a diagnosis of drug-induced delirium. This diagnosis is further supported by the Naranjo Adverse Drug Reaction Probability Scale, which classified the reaction as ‘probable,’ with a score of 6 [11].

Cefepime-induced neurotoxicity is more common in renal impairment due to renal excretion of cefepime [4]. The patient's chronic kidney disease and acute-on-chronic renal dysfunction may have contributed to cefepime accumulation and increased susceptibility to neurotoxicity.

The patient exhibited classic neuropsychiatric manifestations of CIN, including temporal disorientation, visual hallucinations, and agitation. Previous studies, such as those by Payne et al. and Appa et al., have documented similar presentations, often leading to misdiagnosis as a primary psychiatric disorder or an alternative neurological condition [6,10]. This diagnostic challenge underscores the need for heightened clinical suspicion in hospitalised patients receiving cefepime and presenting acute/subacute psychopathological symptoms, especially those with renal impairment [10].

The temporal association between the onset of delirium one day after the first cefepime administration, along with the resolution of symptoms one day after the drug discontinuation, is consistent with the available evidence and supports the diagnosis of CIN. The symptoms typically resolve within a few days of cefepime discontinuation, which was also observed in our patient, reinforcing the likelihood of a causal relationship [6].

This case highlights the need for close monitoring for neurotoxicity, even with renal-based cefepime dose adjustments. Although dose adjustments reduce risk, they do not eliminate it [6]. Previous reviews report CIN cases in patients receiving recommended doses for their renal status [6,10]. Prompt identification and cessation of cefepime are essential to prevent complications. If treatment continuation is necessary, dose adjustment, antiepileptics, benzodiazepines, or haemodialysis may help manage neurotoxicity [12]. A limitation of this report is that electroencephalography and cefepime serum concentrations were not obtained. Therefore, objective confirmation of cefepime-induced neurotoxicity was not available. In addition, detailed serial measurements of renal function at the time of cefepime initiation, symptom onset, and drug discontinuation were not available for retrospective review. Nevertheless, the temporal relationship between cefepime exposure and symptom onset, the absence of acute structural abnormalities on brain imaging, and the complete resolution of symptoms within 24 hours of cefepime withdrawal support cefepime-induced neurotoxicity as the most likely diagnosis. A further limitation of this report is that detailed information regarding cefepime dosing, dosing interval, treatment duration, and renal dose adjustment was not retrievable from the available documentation, which precluded a more comprehensive assessment of the contribution of drug accumulation to the observed neurotoxicity.

Furthermore, cefepime-induced neurotoxicity should be considered in cases of delirium, especially in renal impairment, emphasising early recognition to prevent lasting damage. Further research should focus on risk stratification and safer antibiotic alternatives.

## Conclusions

This case reinforces CIN as an important and potentially reversible cause of delirium, particularly in patients with chronic kidney disease. Although cefepime remains an effective broad-spectrum antibiotic, clinicians should maintain a high index of suspicion for neuropsychiatric adverse effects, even when renal-adjusted dosing is implemented. The rapid onset of confusional symptoms after cefepime initiation and their prompt resolution following drug discontinuation strongly support a causal relationship. Early recognition of cefepime-induced delirium is essential to avoid unnecessary diagnostic procedures, inappropriate psychiatric interventions, and further clinical deterioration. Careful monitoring of mental status in vulnerable patients receiving cefepime may facilitate timely identification and management of this underrecognised adverse drug reaction.
